# Direction of the formation of anterior lumbar vertebral osteophytes

**DOI:** 10.1186/1471-2474-10-4

**Published:** 2009-01-13

**Authors:** Yuichi Kasai, Eiji Kawakita, Toshihiko Sakakibara, Koji Akeda, Atsumasa Uchida

**Affiliations:** 1 Department of Orthopaedic Surgery, Mie University Graduate School of Medicine, 2-174 Endobashi, Tsu city, Mie prefecture, 514-8507, Japan

## Abstract

**Background:**

X-ray images of lumbar degenerative diseases often show not only claw osteophytes, but also pairs of osteophytes that form in a direction away from the adjacent disc. We have investigated the direction of the formation of anterior lumbar vertebral osteophytes across the lumbar vertebrae using a sufficient number of lumbar radiographs, because osteophytes images can provide essential information that will contribute to the understanding of the pathology and progress of lumbar spine degeneration.

**Methods:**

The direction of the formation of 14,250 pairs of anterior lumbar vertebral osteophytes across the adjacent intervertebral discs in 2,850 patients who were all over 60 years old was investigated. Anterior lumbar vertebral osteophytes were distributed into six groups based on the direction of extension of each pair of osteophytes across the intervertebral disc space.

**Results:**

In L1–L2 and L2–L3, the number of patients classified into groups B (the pair of osteophytes extended in the direction of the adjacent disc) and C (almost complete bone bridge formation by a pair of osteophytes across the intervertebral disc space) was larger than that classified into group D (the pair of osteophytes extended in a direction away from the adjacent disc). In L3–L4, L4–L5 and L5-S1, the number of patients in group D was greater than that of patients belonging to groups B and C.

**Conclusion:**

Our study showed that pairs of osteophytes frequently formed in the direction of the adjacent disc in the upper lumbar vertebrae (L1–L2 and L2–L3) and in the direction away from the adjacent disc in middle or lower lumbar vertebrae (L3–L4, L4–L5, and L5-S1).

## Background

Kirkardy-Willis et al. [1] reported that lumbar degenerative diseases began with disc degeneration, and, during a period characterized by the development of different pathologies including disc herniation, spinal instability and spinal canal stenosis, ended with the formation of anterior lumbar vertebral osteophytes [2,3] that would stabilize the spinal column. According to the radiographic Nathan's classification [4] of anterior lumbar vertebral osteophytes, in a claw osteophyte, a bone bridge forms across the intervertebral disc space as a result of the curve and extension of a cranial osteophyte and a caudal osteophyte across the adjacent disc.

Actually, X-ray images of lumbar degenerative diseases often show not only claw osteophytes, but also pairs of osteophytes that form in a direction away from the adjacent disc. The latter type is called traction osteophytes or traction spurs [5,6], and have been described as indicators of intervertebral instability on a plain radiographic image by Macnub et al. [5]. Besides, Pate et al. [7] reported from their studies in 200 cadavers that both claw and traction osteophytes had the same histology, and that traction osteophytes could turn into claw osteophytes during the lumbar degenerative process. In 1998, Heggeness [8] demonstrated that both claw and traction osteophytes formed as a result of the same degenerative process. For a decade after these reports, researchers have no longer paid attention to the types of osteophytes and the direction of their formation. We have investigated the direction of the formation of anterior lumbar vertebral osteophytes across the lumbar vertebrae using a sufficient number of lumbar radiographs, because osteophytes images can provide essential information that will contribute to the understanding of the pathology and progress of lumbar spine degeneration.

## Methods

The subjects were 2,850 patients (1,511 men and 1,339 women) who visited the spine surgery outpatient clinic of our department or affiliated hospitals between April 2006 and March 2007. They were all over 60 years old (mean, 72.1 ± 9.2 years; range, 60–90 years) and underwent plain X-ray examination of the lumbar spine. Lumbar spinal canal stenosis was diagnosed in 1,278, spondylosis deformans in 673, lumbar discopathy in 352, lumbar disc herniation in 187, lumbar spondylolisthesis in 145, and other diseases in 215 patients.

**Figure 1:**
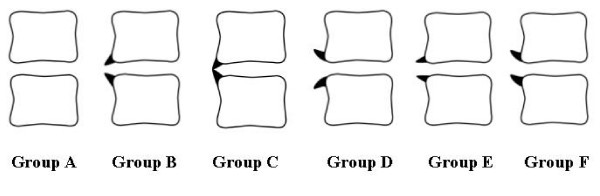
**Classification of the extension direction of each pair of osteophytes across the intervertebral disc space**. Group A: no osteophytes. Group B: the pair of osteophytes extended in the direction of the adjacent disc. Group C: almost complete bone bridge formation by a pair of osteophytes across the intervertebral disc space. Group D: the pair of osteophytes extended in a direction away from the adjacent disc. Group E: the osteophytes extended nearly horizontally to the vertebral body border without closing the intervertebral disc space. Group F: ungroupable

Plain X-ray films of the lumbar spine were obtained in a neutral lateral position and 14,250 pairs of cranial and caudal osteophytes were found across the intervertebral disc space at L1–L2, L2–L3, L3–L4, L4–L5, and L5-S1. Our department and affiliated hospitals used the same X-ray equipment for general purposes (DHF-153H2, manufactured by Hitachi Medico, Tokyo, Japan) in every case. An anterior lumbar vertebral osteophyte should be 2 mm or more in length according to the classification of Macnab et al. [5]. Anterior lumbar vertebral osteophytes were distributed into six groups based on the direction of extension of each pair of osteophytes across the intervertebral disc space as follows (Fig. 1): group A, no osteophytes; group B, the pair of osteophytes extended in the direction of the adjacent disc; group C, there was almost complete bone bridge formation by a pair of osteophytes across the intervertebral disc space; group D, the pair of osteophytes extended in a direction away from the adjacent disc; group E, the osteophytes extended nearly horizontally to the vertebral body border without closing the intervertebral disc space; and group F, ungroupable. Assignment to these groups was performed by two orthopedic surgeons with an agreement ratio of 97.8%, although the judgment by a senior surgeon was accepted if the two surgeons' decisions were not identical.

## Results

**Figure 2:**
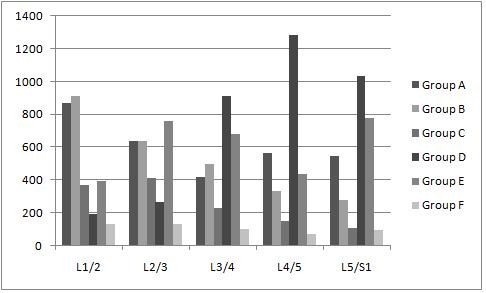
**The number of patients assigned by our classification of anterior lumbar vertebral osteophytes in each intervertebral disc space**.

**Figure 3:**
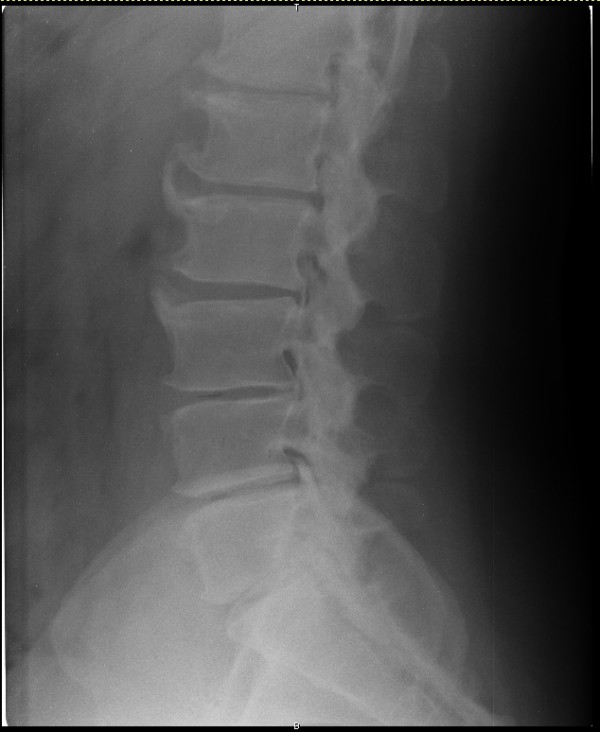
**Representative radiograph of anterior lumbar vertebral osteophytes; 72 years old male**.

The results are summarized in Table 1 and Fig. 2. There were 14,250 pairs of osteophytes in 2,850 patients. As for L1–L2, a total of 837 (32.1%) patients belonged to group A; in the lumbar vertebrae osteophytes formed the least frequently at L1–L2 and the number of patients classified into groups B and C was larger than that of patients classified into group D. In L2–L3, like in L1–L2, the number of patients classified into groups B and C was larger than that of patients classified into group D. In L3–L4, the number of patients in group A was 417 (14.6%); this was the intervertebral disc space at which osteophytes formed most frequently, and unlike in L1–L2 and L2–L3, the number of patients in group D was greater than that of patients belonging to groups B and C. In L4–L5, the number of patients in group D was 1,284 (45.1%); this was the intervertebral disc space at which osteophytes formed most frequently in a direction away from the adjacent disc, and, like in L3–L4, the number of patients in group D was greater than that of patients belonging to groups B and C. In L5-S1 also, like L3–L4 and L4–L5, the number of patients in group D was greater than that of patients belonging to groups B and C. A representative radiograph of anterior lumbar vertebral osteophytes is shown in Fig. 3.

**Table 1:** Overall results

	L1–2	L2–3	L3–4	L4–5	L5-S1
Group A	873 (29.4%)	641 (22.5%)	417 (14.6%)	568 (19.9%)	550 (19.3%)

Group B	916 (32.1%)	638 (22.4%)	501 (17.6%)	336 (11.8%)	279 (9.8%)

Group C	371 (13.0%)	412 (14.4%)	229 (8.0%)	152 (5.3%)	108 (3.8%)

Group D	193 (6.8%)	265 (9.3%)	915 (32.1%)	1284 (45.1%)	1037 (36.4%)

Group E	398 (14.0%)	761 (26.7%)	684 (24.0%)	439 (15.4%)	778 (27.3%)

Group F	135 (4.7%)	133 (4.7%)	104 (3.7%)	71 (2.5%)	98 (3.4%)

Group A: no osteophytes

Group B: the pair of osteophytes extended in the direction of the adjacent disc

Group C: almost complete bone bridge formation by a pair of osteophytes across the intervertebral disc space

Group D: the pair of osteophytes extended in a direction away from the adjacent disc

Group E: the osteophytes extended nearly horizontally to the vertebral body border without closing the intervertebral disc space

Group F: ungroupable

## Discussion

The etiology of anterior lumbar vertebral osteophytes is considered as follows. An increased flexibility between the vertebral bodies due to disc degeneration leads to the production of inhomogeneous mechanical stress on the ossification of bone under the cartilage of the vertebral body; consequently, sclerotic or hyperplastic changes occur on the edge of the vertebral body, leading to the formation of osteophytes [4,9,10]. However, the level of degradation of the intervertebral disc is not always consistent with the degree of formation of osteophytes [8,11], and, like the syndesmophytes described by Schumacher et al. [12], osteophytes may be caused by the ossification of the anterior longitudinal ligament and the annulus fibrosus of the intervertebral disc.

The study of anterior lumbar vertebral osteophytes by O'Neill et al. [9] demonstrated that X-ray examination of the lumbar spine of subjects screened for osteoporosis exhibited an increased frequency of anterior lumbar vertebral osteophytes with aging. Furthermore, Watanabe et al. [13] reported that there was a positive correlation between the size of osteophytes and the age of patients in whom the size of lumbar vertebral osteophytes was measured at the autopsy. These studies indicated that osteophytes were caused by aging changes. On the other hand, anterior lumbar vertebral osteophytes have been reported to occur most frequently at L3–L4 [7,9], and more frequently in men [7,9] and obese patients [14,15] or those with heavy physical activity [9] than in women or other patients. Other studies have revealed that spur formation is showing a tendency to intensify annually in approximately 4% of people [16], and that people with osteophytes are less likely to develop osteoporosis [17].

We reviewed the literature with respect to the direction of spur formation, and found a study by Pate et al. [8] who used 200 cadavers to identify the occurrence rates of claw and traction osteophytes at 2,000 locations in the intervertebral spaces of Th12-L1, L1–L2, L2–L3, L3–L4, and L4–L5. They observed that 182 (91%) of the 200 cadavers had osteophytes in at least one intervertebral space. These spurs were distributed in approximately 48% of the 2,000 locations, with claw osteophytes accounting for about 39% and traction osteophytes accounting for some 9%. The former were dominant in Th12 to L2, while the latter were more evident in L3 to L5. Our results show a similar tendency. Although there is a previous report on the direction of spur formation, we considered that our study was worth the effort because it closely examined a considerable number of cases in terms of the direction of spur formation. We believe the discussion on the direction of spur formation in our study will help researchers in this field explore the causes and meaning of spur formation, and investigate the pathology and progress of lumbar spine degeneration.

The results of this study showed that pairs of osteophytes frequently formed in the direction of the adjacent disc in upper lumbar vertebrae (L1–L2 and L2–L3) and in a direction away from the adjacent disc in middle or lower lumbar vertebrae (L3–L4, L4–L5, and L5-S1). Shao et al. [18] reported that the ventral disc height and lordosis angle were greater in L3–L4, L4–L5, and L5-S1 than in L1–L2 and L2–L3. It was also reported that the degree of disc degeneration tended to be higher in L3–L4, L4–L5, and L5-S1 than in L1–L2 and L2–L3, with intervertebral instability being more frequently observed in the lower lumbar vertebrae. We think that anatomical and biomechanical differences as well as differences in the degenerative process of the upper and lower lumbar vertebrae are among the essential factors that determine the direction of spur formation in intervertebral spaces.

As a limitation of this study, we should mention that the subjects were outpatients of the spine unit of our department. Thus, we are planning to conduct a similar study targeting the general population. In a future study, we will investigate the deformation of multiple intervertebral disc spaces, with regard to biomechanical differences between upper lumbar vertebrae and middle or lower lumbar vertebrae in human lumbar vertebrae of cadavers. We are also planning to investigate the direction of spur formation in a cross-sectional and in a longitudinal study.

## Conclusion

Our study showed that pairs of osteophytes frequently formed in the direction of the adjacent disc in the upper lumbar vertebrae (L1–L2 and L2–L3) and in the direction away from the adjacent disc in middle or lower lumbar vertebrae (L3–L4, L4–L5, and L5-S1).

## Abbreviations

none

## Competing interests

The authors declare that they have no competing interests.

## Authors' contributions

YK and EK drafted the manuscript, did first selection of articles, and assessed the quality of the papers. TS and KA gave important inputs for the methodic part of this paper, assessed the quality of the papers, performed the statistical analysis, and revised the manuscript critically for its content. AU helped to draft and to correct the manuscript. All authors read and approved the final manuscript.

## Pre-publication history

The pre-publication history for this paper can be accessed here:


